# Low-frequency EPR of ferrimyoglobin fluoride and ferrimyoglobin cyanide: a case study on the applicability of broadband analysis to high-spin hemoproteins and to HALS hemoproteins

**DOI:** 10.1007/s00775-022-01948-1

**Published:** 2022-07-08

**Authors:** Wilfred R. Hagen

**Affiliations:** Department of Biotechnology, Delft University of Technology, Delft, The Netherlands

**Keywords:** EPR, Low-frequency, Broadband, Hemoprotein, Myoglobin, HALS

## Abstract

An EPR spectrometer has been developed that can be tuned to many frequencies in the range of ca 0.1–15 GHz. Applicability has been tested on ferrimyoglobin fluoride (MbF) and ferrimyoglobin cyanide (MbCN). MbF has a high-spin (*S* = 5/2) spectrum with ^19^F superhyperfine splitting that is only resolved in X-band along the heme normal. Low-frequency EPR also resolves the splitting in the heme plane. Measurement of linewidth as a function of frequency provides the basis for an analysis of inhomogeneous broadening in terms of *g*-strain, zero-field distribution, unresolved superhyperfine splittings and dipolar interaction. Rhombicity in the *g* tensor is found to be absent. MbCN (*S* = 1/2) has a highly anisotropic low spin (HALS) spectrum for which *g*_*x*_ cannot be determined unequivocally in X-band. Low-frequency EPR allows for measurement of the complete spectrum and determination of the *g*-tensor.

**Graphical abstract:**

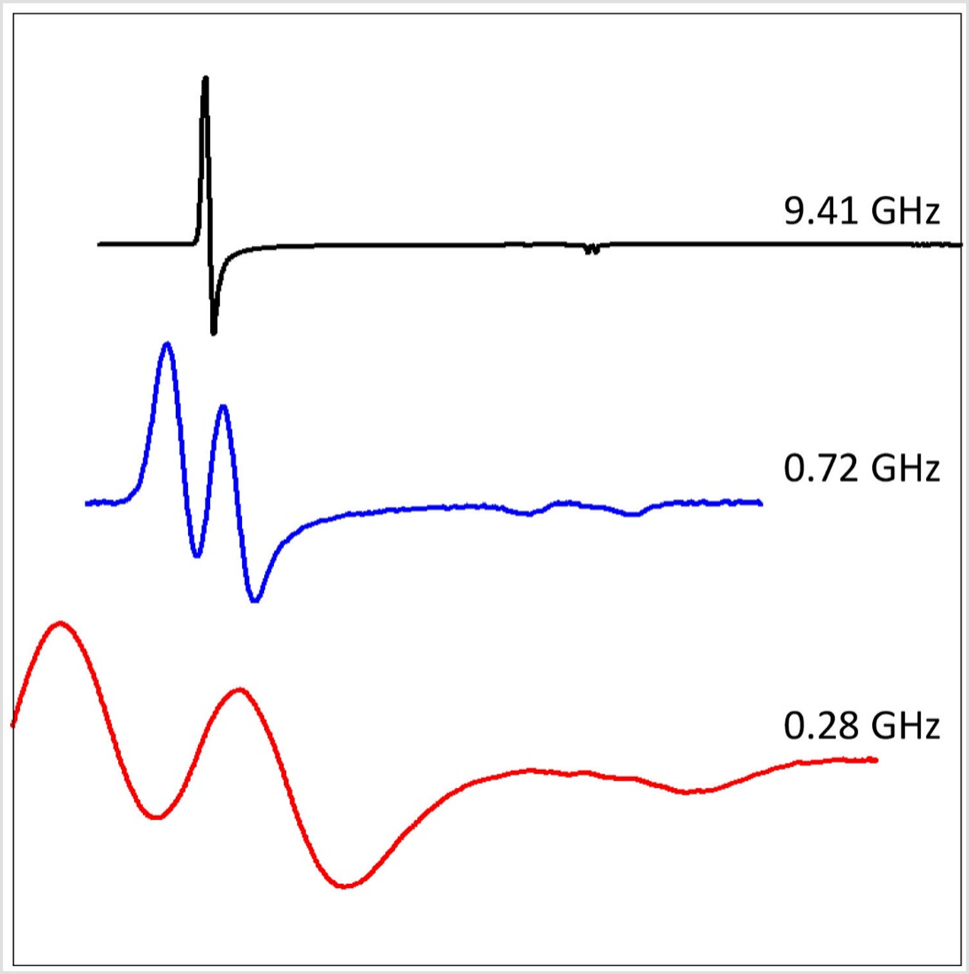

**Supplementary Information:**

The online version contains supplementary material available at 10.1007/s00775-022-01948-1.

## Introduction

EPR spectroscopy holds a prominent position in the toolbox of bioinorganic chemistry for the study of structure and function of transition-metal sites in biomacromolecules. From a practical perspective, the continuous wave (cw) X-band (ca 9–10 GHz) spectrometer has been the common standard for the past six decades. If the problem at hand requires the collection of additional data than the X-band standard can generate, then additional EPR instrumentation is invoked. The latter generally comes under the name of ‘advanced EPR’ [1], and grosso modo encompasses two directions: (i) double-resonance techniques (e.g., ENDOR, ESEEM) for the resolution of small hyperfine interactions between a metal ion and its first and second sphere ligands [2]; and (ii) multi-frequency, in particular high-frequency (and, therefore, high field), EPR for the separation of magnetic field-dependent and field-independent interactions in complex spectra [2–4]. Rather less common is the use of cw frequency sources lower than X-band. This approach typically comes in the form of a separate spectrometer with a monochromatic resonator that operates in a specific frequency band such as S-band (ca 3–4 GHz) or L-band (ca 1–2 GHz) [5–8]. Although the low-frequency spectrometer potentially has the capacity to both resolve hyperfine interactions as well as to provide extra information on electron spin–spin, or zero-field, interactions that may resolve equivocal analyses of X-band data, it has not really raised sustained interest. Relatively low concentration sensitivity is presumably a major determinant in the lack of enthusiasm on the part of the bioinorganic chemist.

As a competitive alternative to the single low-frequency spectrometer, I have developed a new type of machine called a broadband EPR spectrometer as it can be tuned to resonance at many microwave frequencies over a range of up to some seven octaves from above X-band down to well into the sub-gigahertz range [9–11]. Applicability to dilute randomly oriented samples has been illustrated with analysis of site symmetry in a low-symmetry copper complex [10] and recently with analysis of intra- and inter-molecular dipolar heme–heme interaction in low spin ferric cytochromes (*S* = 1/2) [11]. This exploration of usefulness is now extended to the class of high-spin ferric hemoproteins (*S* = 5/2), which pose a very different problem than the previous examples since they exhibit strong zero-field interaction as well as a complex linewidth behavior. Another extension explored here is to HALS (highly anisotropic low spin) hemoproteins, whose *g* tensor is typically only partially detectable in X-band.

**Fig. 1:**
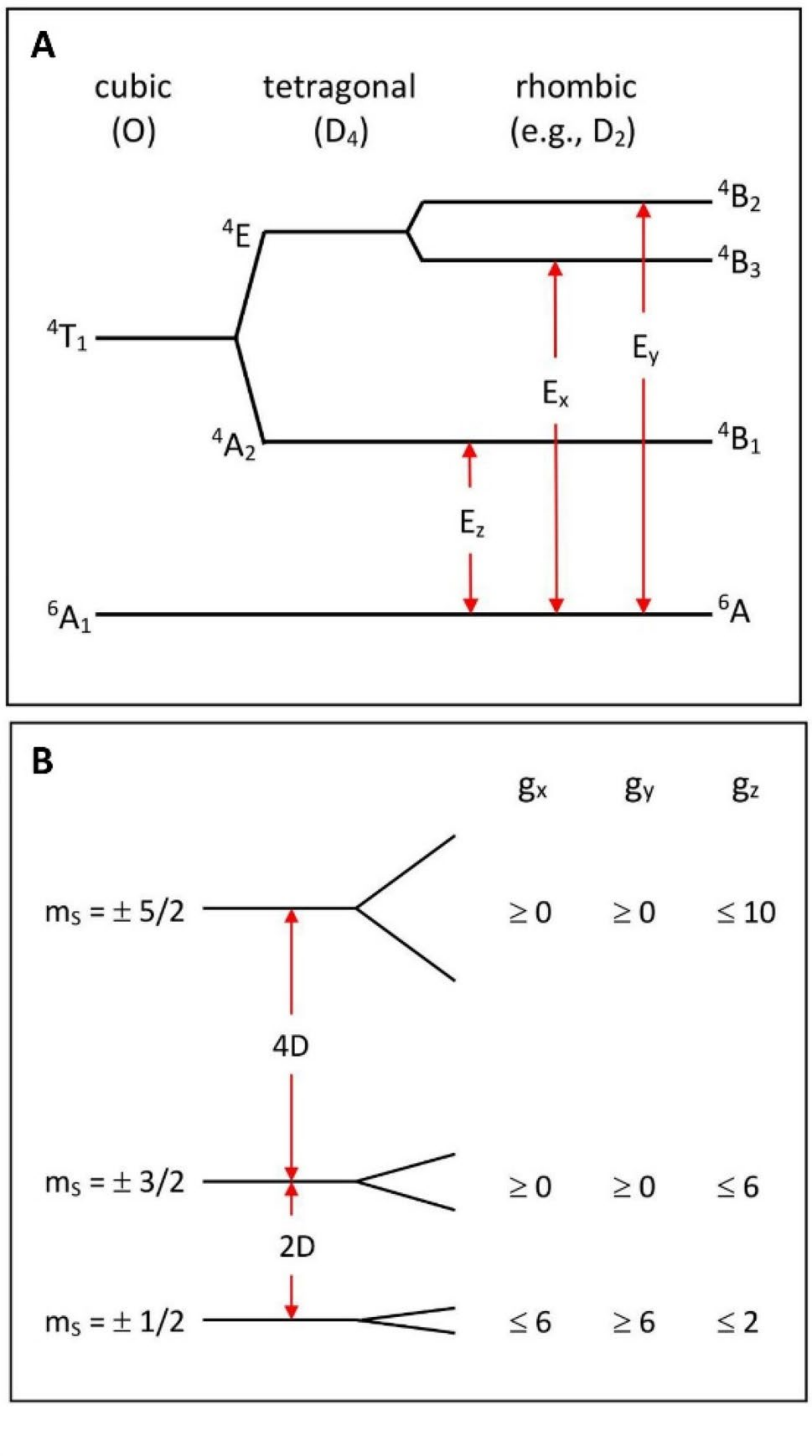
Crystal-field model for high-spin *S* = 5/2 *d*^5^ systems. The model is due to Weissbluth [15] and assumes that the ground spin manifold is determined by interaction of the electronic ground state with the lowest excited state (split by low symmetry) only. In axial symmetry only the EPR transition within the lowest of three Kramers doublets has finite intensity with effective *g* values *g*_⊥_ = 6 and *g*_||_= 2, whereby the real *g* value in all directions is approximately equal to 2 due to quenching of orbital angular momentum in the half-filled *d* shell. A small rhombicity (*E*/*D* > 0) induces a quasi-symmetrical splitting in the *g*_⊥_ value

The EPR spectrum of high-spin ferric hemoproteins is dominated over a very wide frequency range (cf [12–14]) by two leading interactions in the spin Hamiltonian:

$${\mathcal{H}} = D\left[ {S_{z}^{2} - S\left( {S + 1} \right)/3} \right] + E\left( {S_{x}^{2} - S_{y}^{2} } \right) + \beta \cdot B \cdot \hat{g} \cdot S$$

in which the first two terms describe the axial and the rhombic part of the zero-field interaction between the five unpaired electrons of the *S* = 5/2 system and the last term is the electronic Zeeman interaction. The zero-field parameters, *D* and *E*, depend on the low lying crystal-field energies of the rhombic *d*^5^ system (these energies, *E*_*x,y,z*_, are defined in Fig. 1A).

And the relations are [15]:

1 $$D = \frac{{\lambda^{2} }}{10}\left( {\frac{2}{{E_{z} }} - \frac{1}{{E_{x} }} - \frac{1}{{E_{y} }}} \right)$$

2 $$E = \frac{{\lambda^{2} }}{10}\left( {\frac{1}{{E_{x} }} - \frac{1}{{E_{y} }}} \right)$$

in which *λ* is the effective spin–orbit coupling constant of the coordinated Fe(III) ion. With the axial zero-field splitting being at least an order of magnitude greater than the Zeeman interaction at X-band, and with *D* > 0, a spin multiplet with three well-separated Kramers doublets results (Fig. 1B) whose effective *g* values are determined by the rhombicity parameter *E*/*D*, which are readily calculated in the form of rhombograms [16–18]. In practice, the rhombicity of ferric hemes is small and therefore the effective *g* anisotropy for the second and third doublet extends over a nearly infinite field range, and the subspectra have undetectably small amplitude. Only the subspectrum within the *m*_*S*_ =  ± ½ doublet is observed, which behaves as an effective *S* = 1/2 spectrum, albeit with a temperature dependence that deviated from Curie behavior due to partial depopulation of the doublet with increasing temperature. The effective *g* values of this spectrum are given by [19]:

3 $$g_{z}^{{{\text{eff}}}} = g_{z}$$

4 $$g_{y}^{{{\text{eff}}}} = 3g_{y} \left[ {1 - \frac{1}{2}\left( {\frac{h\nu }{D}} \right)^{2} + \frac{4E}{D}} \right]$$

5 $$g_{x}^{{{\text{eff}}}} = 3g_{x} \left[ {1 - \frac{1}{2}\left( {\frac{h\nu }{D}} \right)^{2} - \frac{4E}{D}} \right]$$

in which *g*_*x*_ ≈ *g*_*y*_ ≈ *g*_*z*_ ≈ 2.00 due to the quenching of orbital angular momentum in the half-filled 3d^5^ shell. In such a spectrum the perpendicular feature may be split by a small anisotropy

6 $${\Delta }g_{xy}^{{{\text{eff}}}} \approx 48\frac{E}{D}$$

For ferrimyoglobin fluoride reported *D* values are in the range 5–7 cm^−1^ and *E* is close to zero [13, 14, 20–22]. The example of ferrimyoglobin fluoride was chosen because it poses an interesting spectroscopic puzzle with, in X-band, partially resolved ^19^F superhyperfine splitting, poorly resolved rhombicity in the effective g-tensor due to a small rhombic zero-field interaction, and a complex linewidth pattern, altogether described with the spin Hamiltonian

7 $${\mathcal{H}} = \beta \cdot B \cdot \hat{g} \cdot S + \sum S \cdot \hat{D} \cdot S + \sum S \cdot \hat{A} \cdot I + \beta \cdot B \cdot \widehat{\Delta g} \cdot S + S \cdot \widehat{\Delta D} \cdot S$$

in which the different terms, respectively, stand for (1) electronic Zeeman interaction, (2) zero-field interaction in the *S* = 5/2 multiplet and zero-field interaction from intermolecular dipole–dipole interactions, (3) superhyperfine interactions from the fluoride ligand and from nitrogens and protons in the porphyrin, the axial His ligand and possibly crystal water molecules, (4) *g*-strain broadening from a distribution of *g*-values, and (5) broadening from a distribution in zero-field parameters. Further effective broadening occurs when splittings from the first three terms are poorly resolved.

Furthermore, ferrimyoglobin fluoride has already been extensively studied with frozen-solution EPR [23], single-crystal EPR [21, 24, 25], X- and Q-band ENDOR [22, 26], and high-frequency EPR [13, 14]. We are, therefore, with this system in a good position to rank the value of broadband EPR among ‘advanced EPR’ methods in terms of its potential to provide new, complementary information and/or additional accuracy in the analysis of EPR from high-spin hemoproteins in frozen solution. Results of broadband EPR may also be affirmative of data equally obtainable with other methods, but they may be collected in an easier, cheaper, and faster way.

## Methods

**Fig. 2:**
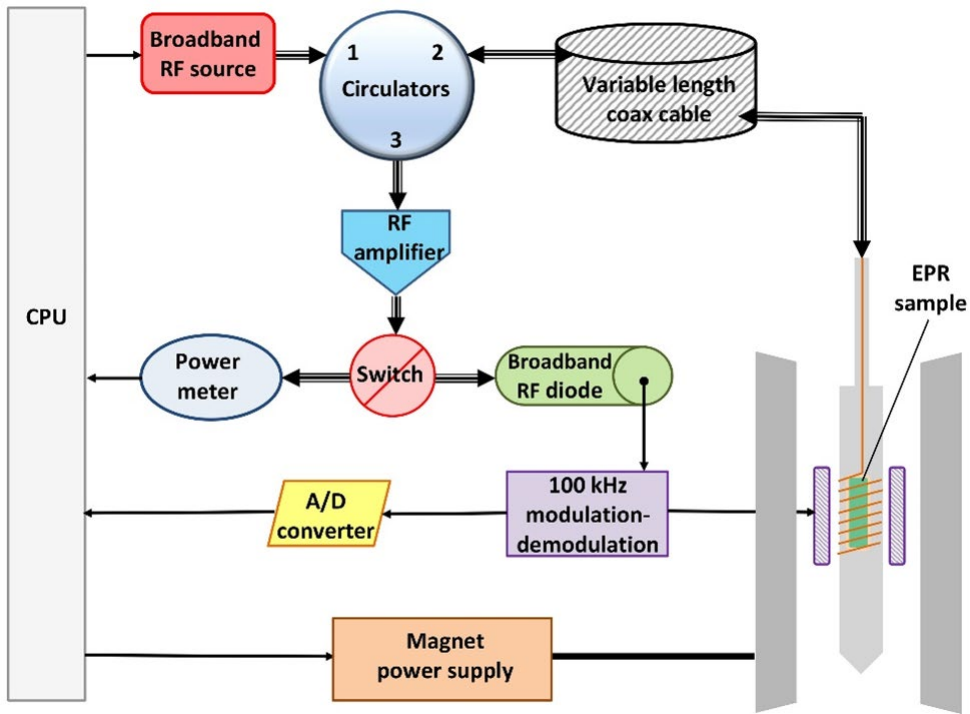
Schematic overview of the broadband EPR spectrometer. The scheme is similar to a previously described one [11] with two modifications. A vector signal transducer (a microwave source and detector) may be replaced with a stand-alone broadband source whereupon detection of the mode pattern of the resonator circuit is taken over by a power meter. See the text for details

The broadband cw EPR spectrometer and peripheral instrumentation and cryogenics have been described in detail in Ref. [11]. A schematic outline is given in Fig. 2 to illustrate two changes that have been implemented since then.

In the previous version a National Instruments Vector Signal Transceiver was used as signal source and monochromatic mode detector up to 6 GHz, whereas higher frequencies were created with a combination of active frequency doublers, and their mode pattern was detected with an 18 GHz power meter and fine-tuned with trombone-type phase shifters. In the present setup the doublers and phase shifters have been omitted and a separate broadband RF source has been added for increased stability above 6 GHz: a 0–15 GHz SynthHD Mini RF Signal Generator by Wildfreak Technologies LLC (New Port Richey, FL, USA). In practice (cf below) sufficient frequency-dependent information is obtained above 6 GHz by recording an X-band spectrum with a conventional spectrometer plus one spectrum around ca 14 GHz using the Mini Signal Generator (maximal power typically 14 dBm at 14 GHz). The Mini Signal Generator may also be used as a cheap (ca 25 ×) alternative for the Vector Signal Transceiver as a < 6 GHz source at the expense of a reduction in signal-to-noise ratio of ca 2 × , and a slowed-down mode-pattern determination.

Horse heart myoglobin was obtained from Sigma-Aldrich (M1882) and used as received. Metmyoglobin fluoride was prepared as a 4 mM solution in 100 mM MES buffer, pH 6.0 plus 100 mM KF. Metmyoglobin cyanide was prepared as a 5 mM solution in 200 mM potassium phosphate buffer, pH 7.4 plus 100 mM KCN.

## Results

### Overview of myoglobin fluoride broadband EPR

**Fig. 3:**
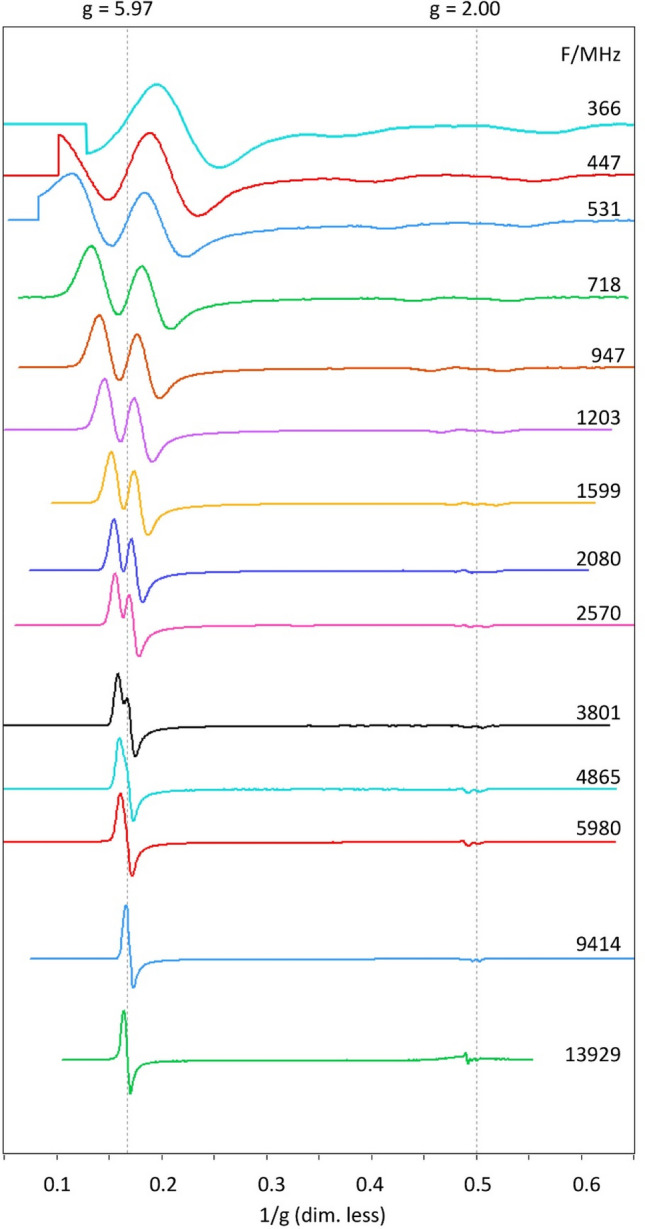
Broadband EPR 2D plot of log(frequency) vs reciprocal *g* values for myoglobin fluoride from 0.37 to 13.9 GHz. Effective *g* values *g*_⊥_ = 5.97 (panel **A**) and *g*_*z*_ = 2.00 (panel **B**) are indicated with vertical dotted lines. EPR conditions: elongation cable length 20 m (0.37–0.95 GHz), 2 m (1.20–2.08 GHz), or 0 m (≥ 2.57 GHz); modulation amplitude, 3 gauss (2 gauss at 0.37 GHz); time per scan, 20 s; averaging 10–60 min; temperature, 9 K

The low-temperature (8–9 K) frozen solution spectrum of ferric myoglobin fluoride at 14 different frequencies from 0.37 to 13.93 GHz is presented in Fig. 3.

The 9.14 GHz spectrum has been measured in a regular X-band machine. At the lowest frequencies a part of the spectrum near zero field is not recordable due to the remnant field of ca 33 gauss of the electromagnet. Note also that at the lowest frequencies the ^19^F parallel hyperfine pattern is no longer symmetric around *g* = 2 because the hyperfine interaction becomes comparable in magnitude to the electronic Zeeman interaction. In terms of the common perturbation-theory analysis this means that the hyperfine interaction is no longer a linear perturbation as second- and higher-order terms become important. At the lowest employed frequency of 0.28 GHz, this even leads to a convolution of the second perpendicular hyperfine line with the first parallel hyperfine line (Fig. S1).

The spectrum is disturbed near *g* = 2 by a small contamination with a copper species with integrated intensity < 1% of Mb (Fig. S2). At the highest frequency of 13.93 GHz the copper signal is almost fully separated from the ^19^F doublet, but when hyperfine interactions become more dominant with decreasing frequency the copper perpendicular feature increasingly interferes with the MbF spectrum until, at the lowest frequencies, it becomes too broad to be detected. This is an illustration of the possibility to minimize interference of overlapping spectra by a judicious choice of the microwave frequency. Note that in the X-band spectrum at 9.14 GHz the copper signal is largely suppressed by saturation as the X-band cavity allows for higher effective power levels than the broadband wire microstrip resonator (cf Fig. S2).

### Resolution of ^19^F hyperfine splitting at low microwave frequency

**Fig. 4:**
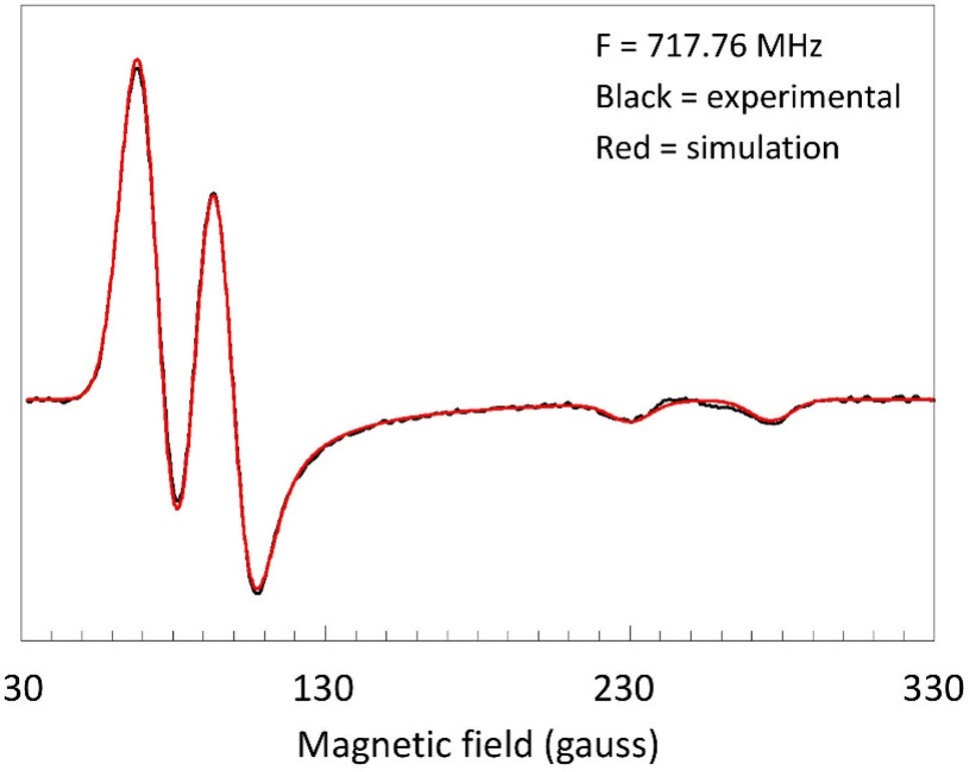
Experimental (black) and simulated (red) spectrum of myoglobin fluoride at 0.17 GHz. The simulation assumes an axial effective *g* tensor (5.97, 2.00) an axial ^19^F superhyperfine tensor (23.8 and 45.2 gauss) and a Gaussian lineshape of isotropic width (16 gauss FWHH)

The most conspicuous feature of the broadband experiment is a complete resolution of the ^19^F splitting at lower frequencies, where in X-band this resolution is limited to the parallel direction. The spectrum at 0.72 GHz is readily reproduced by simulation under the simple model of an axial effective *g*-tensor, an axial ^19^F hyperfine tensor, and a Gaussian lineshape of isotropic width (Fig. 4).

The simulation parameters *A*_||_= 45.2 gauss (127 MHz) and *A*_⊥_ = 23.8 gauss (67 MHz) are essentially identical to the values of *A*_||_= 127 MHz and *A*_⊥_ = 64 MHz previously obtained for a sample of horse MbF at pH 6.0 in 60% glycerol with X-band pulse ENDOR at 2 K [22].

*A*_||_ is resolved at all frequencies in Fig. 3, and is readily determined from the standard cw X-band spectrum. The *A*_⊥_ value could only be determined in double resonance, and is now confirmed in the broadband experiment. The latter can thus be considered as a possible alternative to ENDOR for the full determination of hyperfine interaction that is only partially resolved in cw X-band. The broadband experiment may find preference in being cheaper (hardware and helium consumption), as well as being simpler experimentally. On the other hand, ENDOR also allows for the determination of ^19^F hyperfine splitting for intermediate orientations, and its analysis has indicated minor non-collinearity between the effective *g*-tensor and the hyperfine tensor in the form of a slight tilting of the hyperfine z axis away from the effective *g*-tensor *z* axis over some 5° [22]. Using a simulator for triclinic symmetry [10] I find that such a small deviation would not significantly affect the low-frequency cw spectrum in Fig. 4, and so the effect is not resolvable in cw.

### Lineshape broadening as a function of frequency

**Fig. 5:**
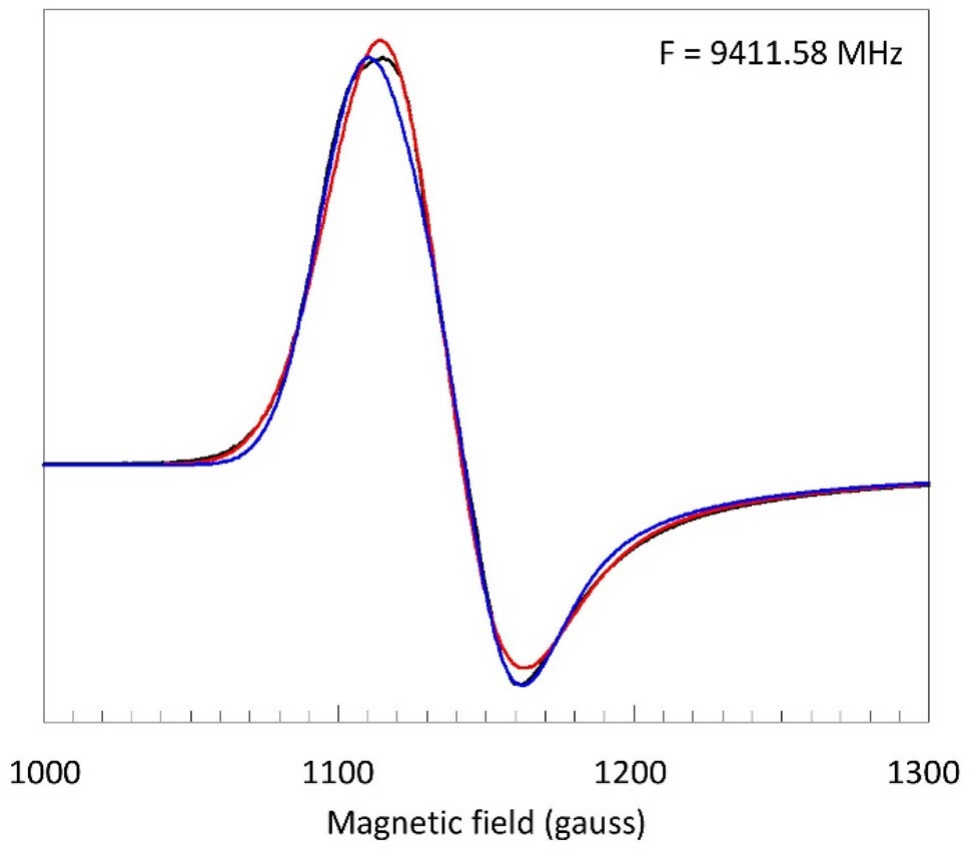
Attempts to simulate the perpendicular feature in X-band based on a simple Gaussian line shape plus ^19^F splitting only. The black trace is the experimental spectrum of myoglobin fluoride; the blue trace has *A*_⊥_ = 24.7 gauss and *W*_⊥_ = 18.5 gauss; the red trace has *A*_⊥_ = 0 and *W*_⊥_ = 25 gauss. The simulations show that the unresolved ^19^F superhyperfine splitting in the perpendicular spectrum cannot be determined from the X-band spectrum. EPR conditions: modulation amplitude, 5 gauss; microwave power, 2 mW; temperature, 8.2 K

An early X-band single-crystal study on sperm whale MbF indicated a slight rhombicity in the effective *g*-tensor (*g* = 6.02, 5.92, 2.006) as well as in the ^19^F hyperfine tensor (*A* = 21.5, 23.5, 43.0 gauss) [24]. However, in a later X-band study on frozen solution of horse MbF such rhombicity was not resolved [22]. It was also concluded that a small *Δg*_⊥_ ≈ 0.1 could have no influence on the ENDOR analysis, and that *E* = 0 [22]. In the absence of rhombicity the simple axial model used for the simulation of the 0.72 GHz spectrum in Fig. 4 should then also be applicable to the X-band spectrum, and so the question arises if, when broadband data are not available, the ^19^F *A*_⊥_ value could not have been estimated from a simulation of the low-field part of the X-band spectrum. The answer is in Fig. 5.

Under the simple model of Fig. 4 a simulation of the 9.4 GHz X-band spectrum with *A*_⊥_ ≈ 24 gauss is not significantly better than one assuming *A*_⊥_ = 0, or, for that matter, with any value in between 24 and 0 gauss. In conclusion, the perpendicular part of the X-band cw-EPR spectrum does not provide resolvable information on the ^19^F hyperfine interaction, which may be taken as an illustration of the frequent impossibility to unequivocally interpret EPR data taken at a single frequency only. An explanation for this lack of resolution is suggested by the magnitude of the linewidths required for the optimized fits in Fig. 5. Their values are at least 2.5 times greater than the value used in the 0.72 GHz simulation in Fig. 4. We will now seek to explain this observation by a study of linewidth as a function of microwave frequency in absence of lifetime broadening and modulation broadening.

**Fig. 6:**
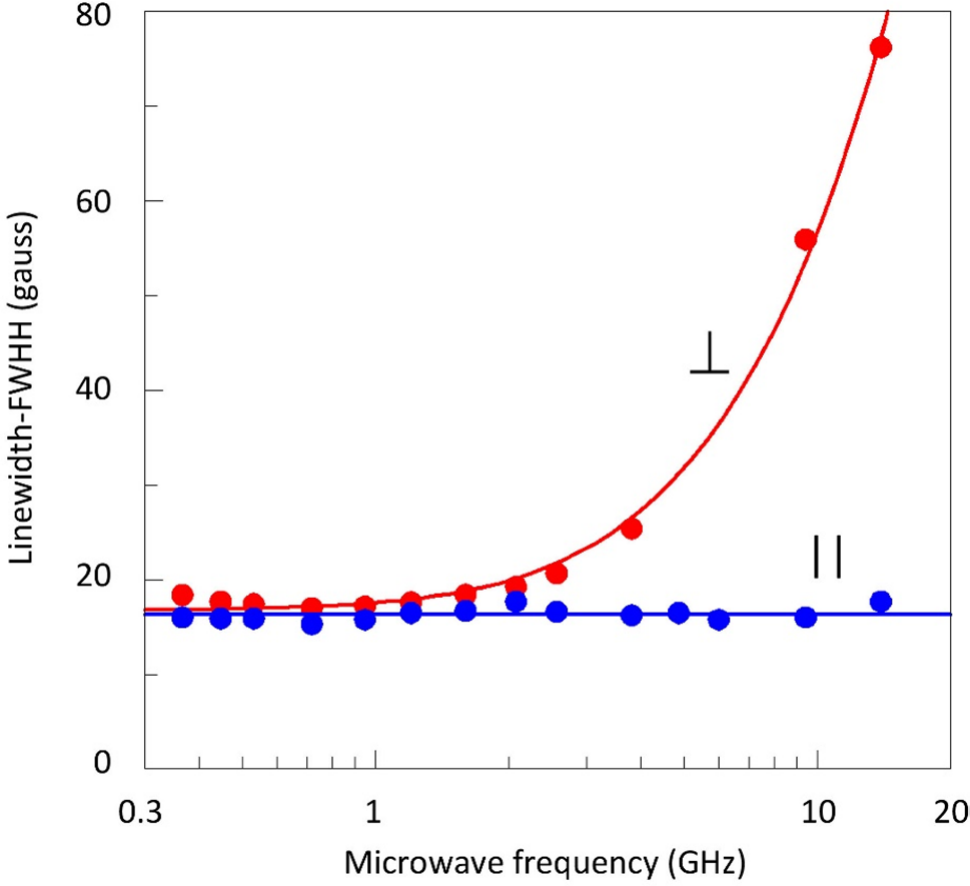
Parallel (blue) and perpendicular (red) linewidth as a function of microwave frequency in the EPR spectrum of myoglobin fluoride. The parallel linewidth (FWHH) was determined by simulation of the high-field peak of the doublet around *g*_*z*_; its constancy indicates only frequency-independent broadening contributions. The perpendicular linewidth was measured as field distance of the peak and negative peak of the derivative-like feature around *g*_⊥_; the observed pattern indicates frequency-independent and frequency-dependent contributions to the broadening

Since the 0.72 GHz spectrum of Fig. 4 was found to be reproducible, by simulation, assuming a single Gaussian lineshape only, we can follow the frequency dependence of its width by measuring the peak-to-peak distance of the perpendicular feature and the full width at half height of the absorption-shaped parallel feature. Since the latter shows interference with a contaminating copper signal, we determine the width with a fit of the high-field line of the doublet to a Gaussian. The results of this broadband linewidth experiment are in Fig. 6.

At low frequency the perpendicular width is found to be independent of the frequency. Around 2 GHz a second broadening mechanism sets in, which is now linear in the frequency. A further increase leads to a frequency range in which the resolution of the ^19^F hyperfine splitting is lost and the individual linewidth cannot be determined. From approximately X-band onwards the linewidth is dominated by frequency-dependent terms, and the constant, low-frequency contribution has become insignificant. The data can be simulated as the convolution of two independent contributions, one, *W*_0_, independent of the frequency *F*, and a second, *W*_*F*_, linear in the frequency. Since the contributions are unrelated, they can be added in quadrature:

8 $$W\left( F \right) = \sqrt {W_{0}^{2} + \left( {W_{F} \times F} \right)^{2} }$$

By contrast, the width of the parallel doublet lines is constant over the full frequency range of Fig. 6. We can, therefore, study the nature of the broadening at any one of the used frequencies. But first, with the data of Fig. 6 in hand, we return to the analysis of the X-band perpendicular feature.

### The X-band spectrum revisited

**Fig. 7:**
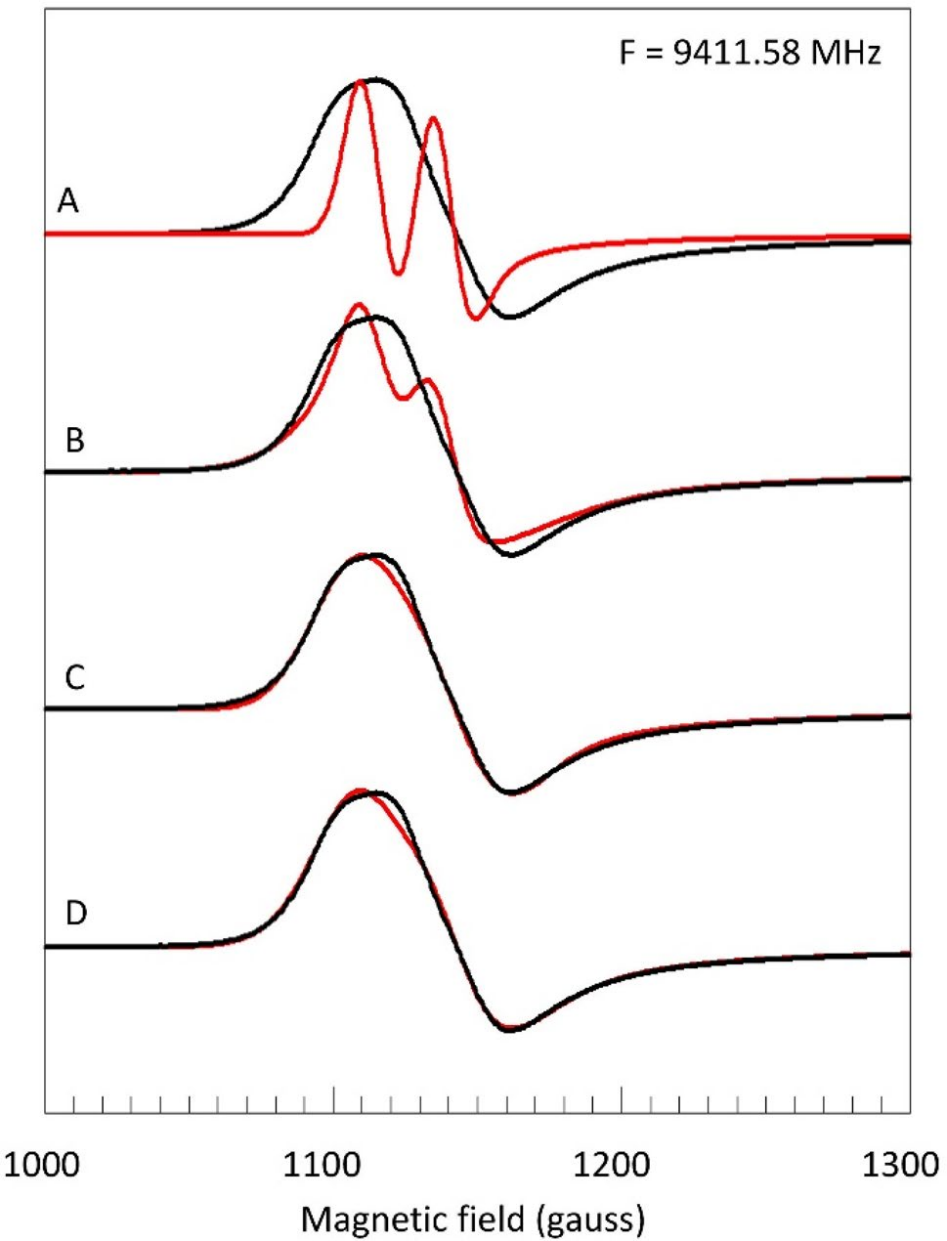
Analysis of the perpendicular feature in the X-band spectrum of myoglobin fluoride based on multiple broadening contributions. The experimental spectrum (black) is as in Fig. 5. The simulation (red) in trace A uses the ^19^F superhyperfine interaction as determined in Fig. 4 and *g*_⊥_ = 1.9925; *D* =  + 5.5 cm^−1^; *E* = 0. In trace B-D, respectively *E*-strain (*σ*_*E*_ = 0.048 cm^−1^; *g*_⊥_ = 1.992), *g*-strain (*σ*_*g*_ = 0.1; *g*_⊥_ = 1.9927) or their combination (*σ*_*E*_ = 0.03 cm^−1^; *σ*_*g*_ = 0.075; *g*_⊥_ = 1.993) is added

Let us then redo the analysis of the perpendicular part of the X-band spectrum, but now we start with the fixed values of *A*_⊥_ = 23.4 gauss and linewidth (FWHH) *W*_⊥_ = 16 gauss as determined in Fig. 4. A simulation based only on these parameters of course gives a poor fit to the X-band spectrum (Fig. 7, trace A) because the frequency-dependent broadening (Fig. 6) is missing.

There is a number of candidates for this contribution: (i) *g*-strain, (ii) an unresolved splitting from a finite *E* ≠ 0, and (iii) a distribution in the zero-field interaction, in particular a Gaussian distribution in *E* (*E*-strain). This latter is based on the model in Fig. 1: when the energies *E*_*x*_ and *E*_*y*_ in Eq. (2) are taken to be distributed by two equal, but independent, Gaussian distributions then the resulting distribution in *E* is also essentially a Gaussian for small standard deviations of *E*_*i*_.

Inclusion of an *E*-distribution around < *E* = 0 > starts to fill up the experimental envelope with good fit especially in the outer wings, but otherwise with insufficient overall broadening (trace B). However, a much better fit is obtained when a co-linear (i.e., *Δg*_*ij*_ = 0) g strain is taken as the cause of frequency-dependent broadening, although now a slight misfit is seen in the outer wings (trace C).

Combining the two frequency dependent broadening mechanisms of *g*-strain and *E*-strain gives a further slight improvement of the fit (trace D). The maximal width of the *E*-distribution is now limited to a standard deviation of *σ*_*E*_ ≤ 0.03 cm^−1^ and the contributions from *g*-strain and *E*-strain to the overall FWHH linewidth are of comparable magnitude, that is circa 30–35 gauss each. Adding a finite value for < *E* > does not in any way improve the simulations (not shown), so the *E*-distribution is essentially centered around zero with an upper limit of < *E* >  ≤ 0.003 cm^−1^. In conclusion, only after the analysis of a low-frequency (0.72 GHz) spectrum that was chosen from a broadband data set, we have been able to deconvolute all contributions to the perpendicular feature of the X-band spectrum as encompassing two dominant frequency-dependent terms, *g*-strain and *E*-strain, of comparable strength (both circa 32 gauss), followed by a frequency-independent broadening from unresolved ^19^F hyperfine splitting (23.4 gauss), and completed by a frequency-independent term (circa 16 gauss) of yet to be determined (see below) origin. Concerning the zero-field splitting parameters, *E* is essentially zero, and the value of *D* is based on literature [13–15, 21, 22] and undetermined in this experiment.

### Analysis of the frequency-independent broadening

We have seen that line broadening in the perpendicular direction, that is in the porphyrin plane, becomes frequency independent at low frequencies (Fig. 6), however, broadening in the parallel direction, that is along the normal to the heme plane, is completely independent of the frequency over the whole range of used frequencies. There are no significant contributions from distributed zero-field parameters nor from *g* strain. This observation raises the question: what are the contributions to the parallel line width and to what extent can they be determined from an analysis of the cw EPR spectrum? Since the broadening is identical at all frequencies, we can conveniently choose to analyze the parallel feature recorded in X-band.

**Fig. 8:**
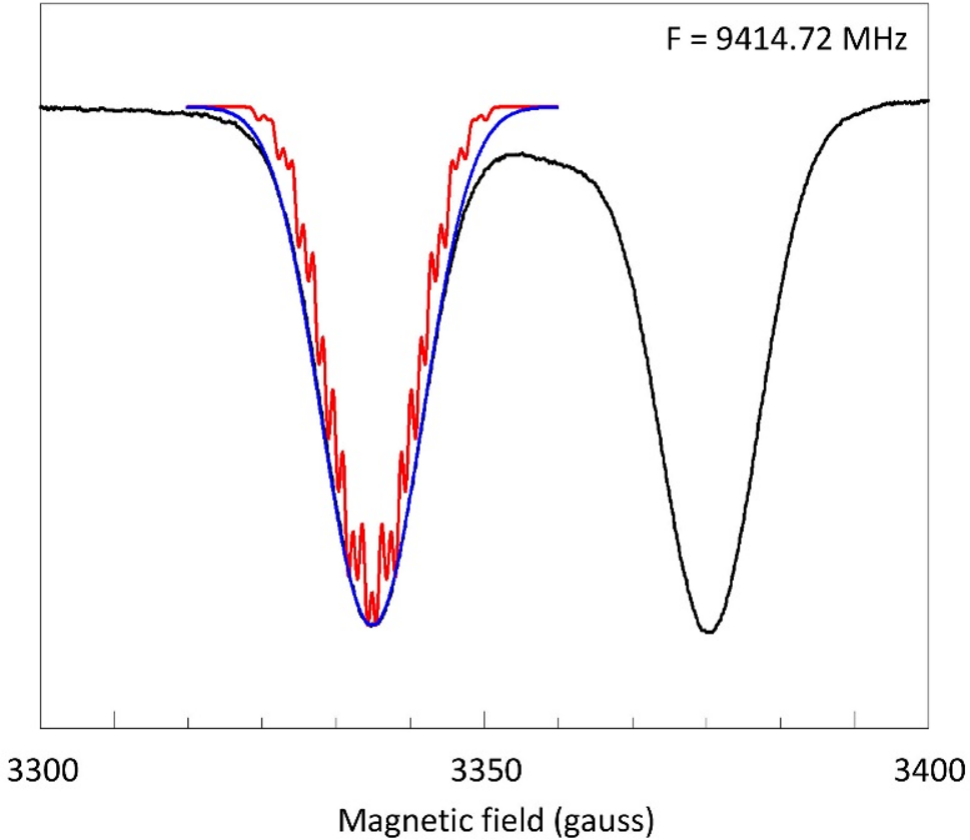
Analysis of inhomogeneous broadening in the EPR parallel feature of myoglobin fluoride. The low-field peak of the ^19^F doublet in X-band is simulated (red) based on ^14^N and ^1^H superhyperfine splittings as reported from ENDOR measurements (see text), *g*_||_= 2.0035, and a linewidth of 0.5 gauss. Increasing the linewidth to 8 gauss affords the blue simulation

ENDOR studies on frozen solutions of myoglobin fluoride have resolved ^14^N (*I* = 1) and ^1^H (*I* = 1/2) interactions in the parallel direction (but not in the perpendicular direction). Magnitudes of the hyperfine splittings are 2.73 gauss (7.65 MHz), on average, for the four porphyrin nitrogens and 3.73 gauss (10.5 MHz) for the *δ*-N for the proximal histidine ligand [26], 0.29 gauss (0.81 MHz), on average, for the four meso protons for the porphyrin ring and 0.45 gauss (1.26 MHz) for the *δ*-N proton of the proximal histidine [27], and 1.36 gauss (3.81 MHz) for the *δ*-N proton of the distal histidine that is hydrogen bonded to the fluoride ligand [22]. Using these values to construct a superhyperfine pattern in the parallel direction can be seen to almost ‘fill up’ one of the ^19^F hyperfine lines at *g*_*z*_ (the red trace in Fig. 8).

The actual resolution of this pattern is likely to be significantly less than in this simulation since it ignores inequivalence in the splitting from the four different tetrapyrrole nitrogens (which is not resolved in ENDOR from frozen solutions [26], but which is observed in ENDOR on a single crystal of myoglobin fluoride [28]) and form the four meso protons. Quadrupole splitting from the nitrogen ligands is also ignored. Furthermore, dipolar broadening from intermolecular interaction is expected to be present as previously observed in broadband EPR of low-spin hemoproteins [11]. As can be seen in Fig. 8, additional, independent, broadening mechanisms are required with an overall line width of some 8 gauss to fit (blue trace) the experimental spectrum.

The following conclusions can be drawn: (1) Although superhyperfine interactions from nitrogens and protons form the major contributions to the parallel line width, their actual magnitudes are not resolved in broadband EPR; (2) Intermolecular dipolar broadening contributes maximally 8 gauss to the overall line width at *g*_*z*_. In fact, since dilution of the protein has no measurable effect on the *g*_*z*_ line (Fig. S3) the dipolar contribution is likely to be significantly less than 8 gauss; (3) Since simulation of the 0.72 GHz spectrum (Fig. 4) requires an isotropic (and frequency-independent) line width, the superhyperfine interaction pattern along *g*_*xy*_ is apparently similar to that along *g*_*z*_.

### Broadband EPR of the HALS hemoprotein Mb-CN

In contrast to the multiple-factored broadening of X-band spectra from high-spin ferric hemoproteins (Figs. 6, 7, 8) the X-band EPR of mononuclear low-spin hemoproteins is completely dominated by *g* strain only [1, 11, 29]. Since spectra are recorded as magnetic–field scans, that is on a reciprocal *g*-value scale, the high-field peak (by convention labeled as *g*_*x*_) is usually the broadest one, and is hence more difficult to detect than the rest of the spectrum. An extreme example of this broadening is found in the sub-class of HALS (highly anisotropic low-spin) hemoproteins, which typically have *g*_*x*_ ≤ 1 and thus exhibit a *g*_*x*_ peak that may easily be broadened beyond detection and/or may be centered at a field value beyond the magnetic-field range of X-band spectrometers. The *g*_*x*_ value of HALS spectra is therefore commonly calculated, rather than measured, on the basis of a simplified ligand-field theory known as the *t*_2*g*_ hole model due to Griffith [30, 31].

**Fig. 9:**
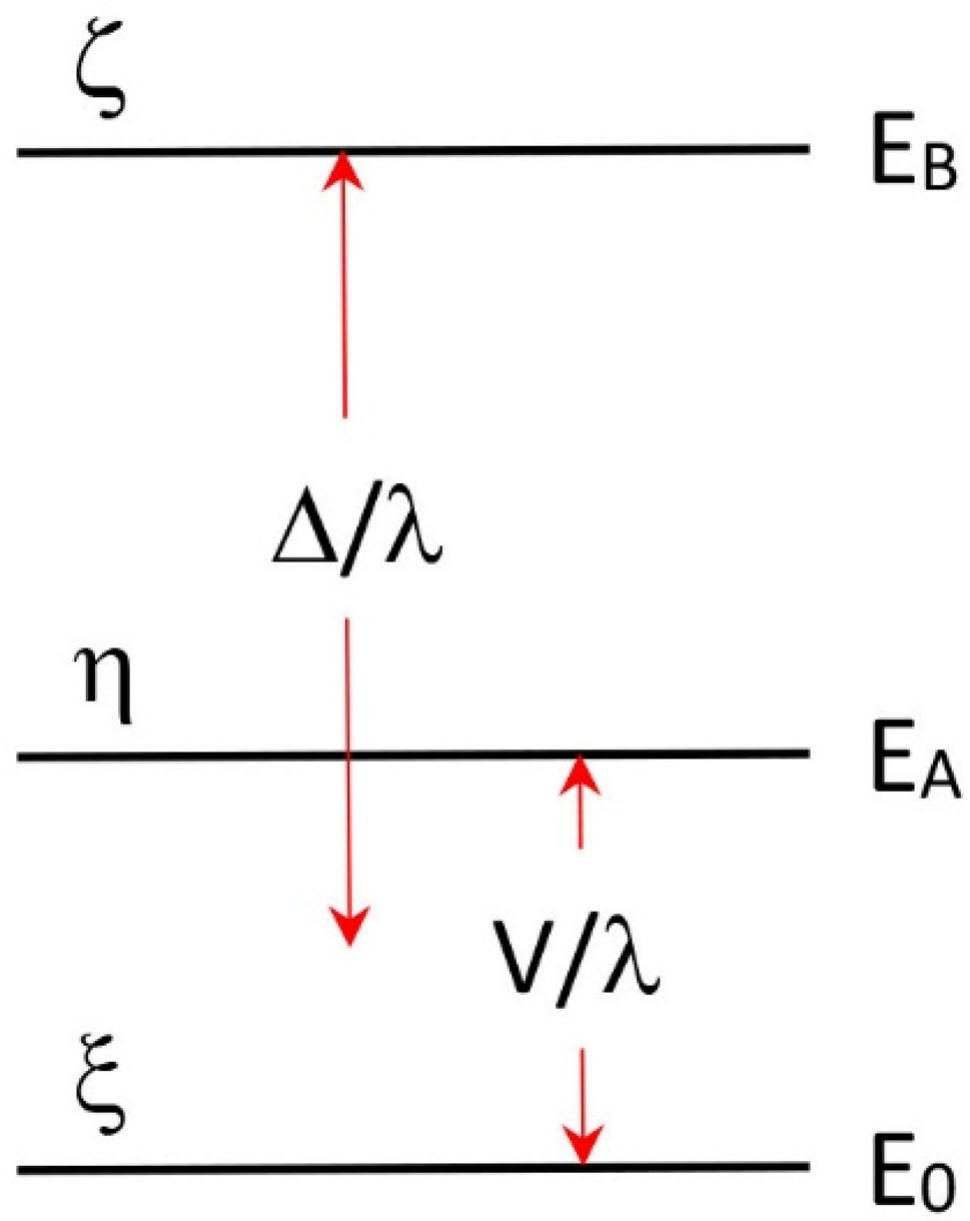
The rhombic energy scheme for low-spin 3*d*^5^. The symbols *ξ*, *η*, and *ζ* are the three single electron hole configurations. *Δ*/*λ* is the axial crystal-field splitting and *V*/*λ* is the rhombic splitting, where *λ* is the spin–orbit coupling constant of the Fe(III) ion

In an octahedral crystal field the five *d* electrons of the low-spin *d*^5^ configuration occupy the *d*_*xy*_, *d*_*xz*_, and *d*_*yz*_ orbitals, which have *t*_2*g*_ symmetry in the group *O*_*h*_. In hemoproteins the symmetry is lowered since the axial ligands differ from the equatorial ones and also due to porphyrin deformation in the asymmetric protein. Three closely spaced (≈ 1000 cm^−1^) electron configurations ensue, which can be considered as an electron hole in each of the *d*_*ij*_ orbitals. With the three hole configurations labeled as *ξ*, *η*, and *ζ,* the energy scheme given in Fig. 9 is usually drawn in ‘rhombic’ symmetry [30, 32]

in which *Δ*/*λ* is the tetragonal splitting in units of *λ*, the spin orbit coupling constant, and *V*/*λ* is the rhombic splitting. The hole states *ξ*, *η*, and *ζ* further mix through spin–orbit coupling to result in three Kramers doublets, the lowest of which gives rise to the detected EPR spectrum. Taylor has shown that the tetragonal and rhombic ligand field splittings are readily obtained from the *g* values of the lowest Kramers doublet:

9 $$E_{A} = \frac{{g_{x} }}{{g_{z} + g_{y} }} + \frac{{g_{y} }}{{g_{z} - g_{x} }}$$

10 $$E_{B} = \frac{{g_{x} }}{{g_{z} + g_{y} }} + \frac{{g_{z} }}{{g_{y} - g_{x} }}$$

with *Δ*/*λ* = *E*_*B*_ – (*E*_*A*_/2) and *V*/*λ* = *E*_*A*_ [32]. Subsequently, numerous attempts have been made to relate the values of *Δ* and *V* to molecular structural and biological functional properties (e.g., Refs. [33–36]).

To extend these type of studies to HALS hemoproteins (e.g., Refs. [34, 37, 38]) the hole model has been further simplified [31] with the restrictions: (1) the orbital reduction factor, *k*, is exactly equal to unity, that is the electron orbitals are pure *d* orbitals and so the coordination bond is assumed to be purely ionic; and (2) the sum of the squares of the *g* values is assumed to be equal to its theoretical maximum for *k* = 1, namely

11 $$S_{g} = g_{x}^{2} + g_{y}^{2} + g_{z}^{2} = 16.0$$

With the two low-field *g*-values measured, the *g*_*x*_ can be calculated from Eq. (11) [39], and the tetragonal and rhombic crystal-field parameters are obtained from the complete *g*-value set, Eqs. (9) and (10). This is clearly a last resort for which justification is hard to find. Covalency (*k* < 1) is the rule in the iron coordination in low-spin hemoproteins, e.g., as evidenced by the measurement of finite ligand hyperfine splittings in ENDOR spectroscopy [40]. Also, there is no a priori reason why the sum in Eq. (11) should equal its maximum of 16; it hardly ever is for systems for which all *g* values are measurable. To avoid any circular reasoning the actual determination of *g*_*x*_ values is desirable. It turns out that low-frequency EPR provides this possibility. Previous attempts to pin down *g*_*x*_ values based on the special techniques of electron spin echo field sweeps or electron spin transient mutation appear to have been unsuccessful [41].

**Fig. 10:**
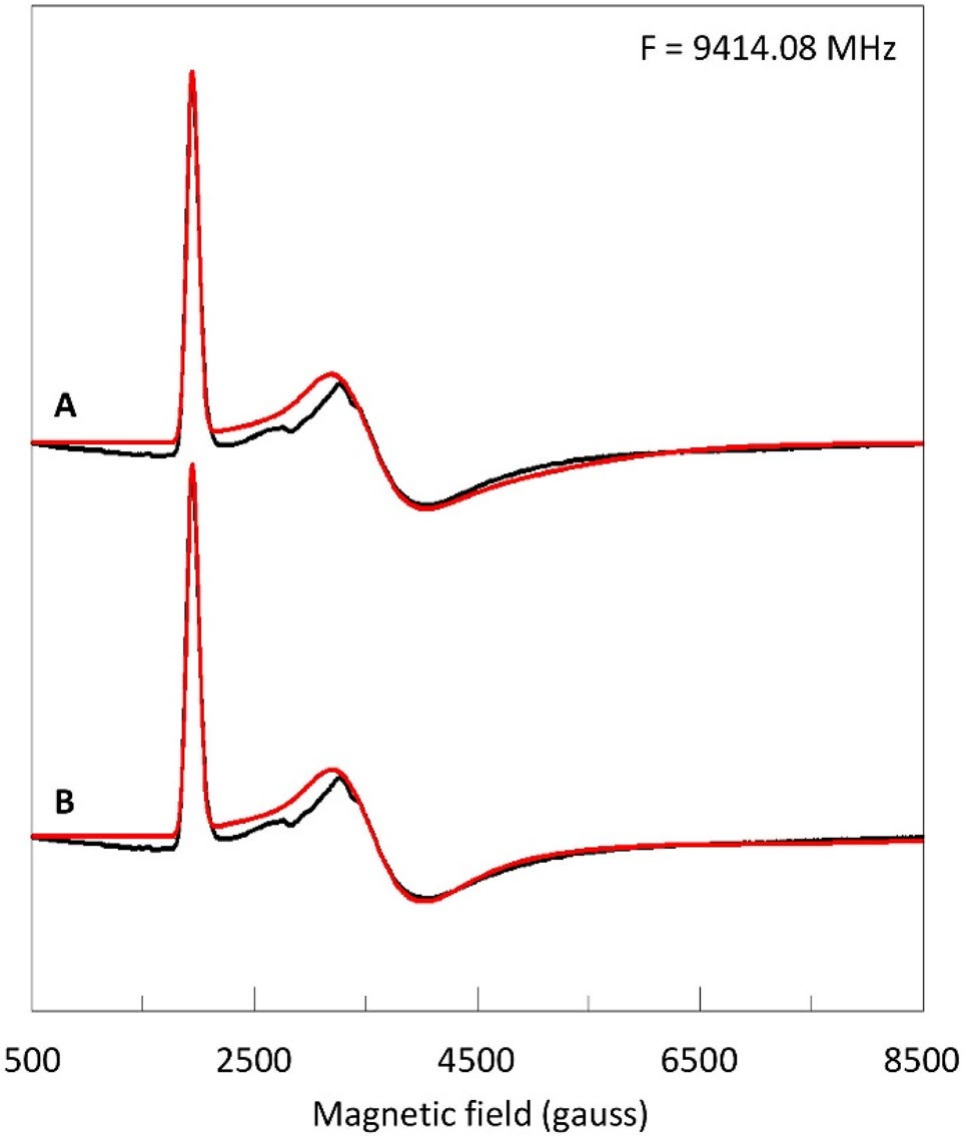
X-band HALS EPR spectrum of myoglobin cyanide. The simulation (red) in trace A is based on *S*_*g*_ = 16.0; the simulation in trace B has *S*_*g*_ < 16. The analysis shows that the high-field *g* value, *g*_*x*_ is undeterminable in X-band. EPR conditions: modulation amplitude, 16 gauss; microwave power, 50 mW; temperature, 13 K. Spectrometer background signals have been removed using the spectrum of a water baseline

The problem of *g*-value determination in HALS spectra is illustrated in Fig. 10 in which a simulation is attempted of the X-band spectrum of myoglobin cyanide.

HALS spectra are very wide and usually extend beyond the maximal field of X-band spectrometers. As a consequence, the spectral amplitude is modest even for solutions of high concentration, and the spectra are typically disturbed by the baseline not being straight. Measuring experimental peak and zero crossing gives *g*_*z*_ = 3.46 and *g*_*y*_ = 1.86. In a simulation these values slightly shift to *g*_*z*_ = 3.45 and *g*_*y*_ = 1.83, and from Eq. (11) we then obtain *g*_*x*_ = 0.87 (*B*_*x*_ = 7731 gauss). These *g* values were used to produce the simulation (red) in Fig. 10A. The *g*-strain simulator has been described before [11, 18]. Lowering the *g*_*x*_ to 0.2 (*B*_*x*_ = 33,631; *S*_*g*_ = 15.3) gives an almost identical (in fact slightly better) fit to the experimental spectrum. It is clear that the value of *g*_*x*_ cannot be determined from the experiment in Fig. 10, and, furthermore, that no evidence is obtained that would support the assumption of *S*_*g*_ = 16.0.

**Fig. 11:**
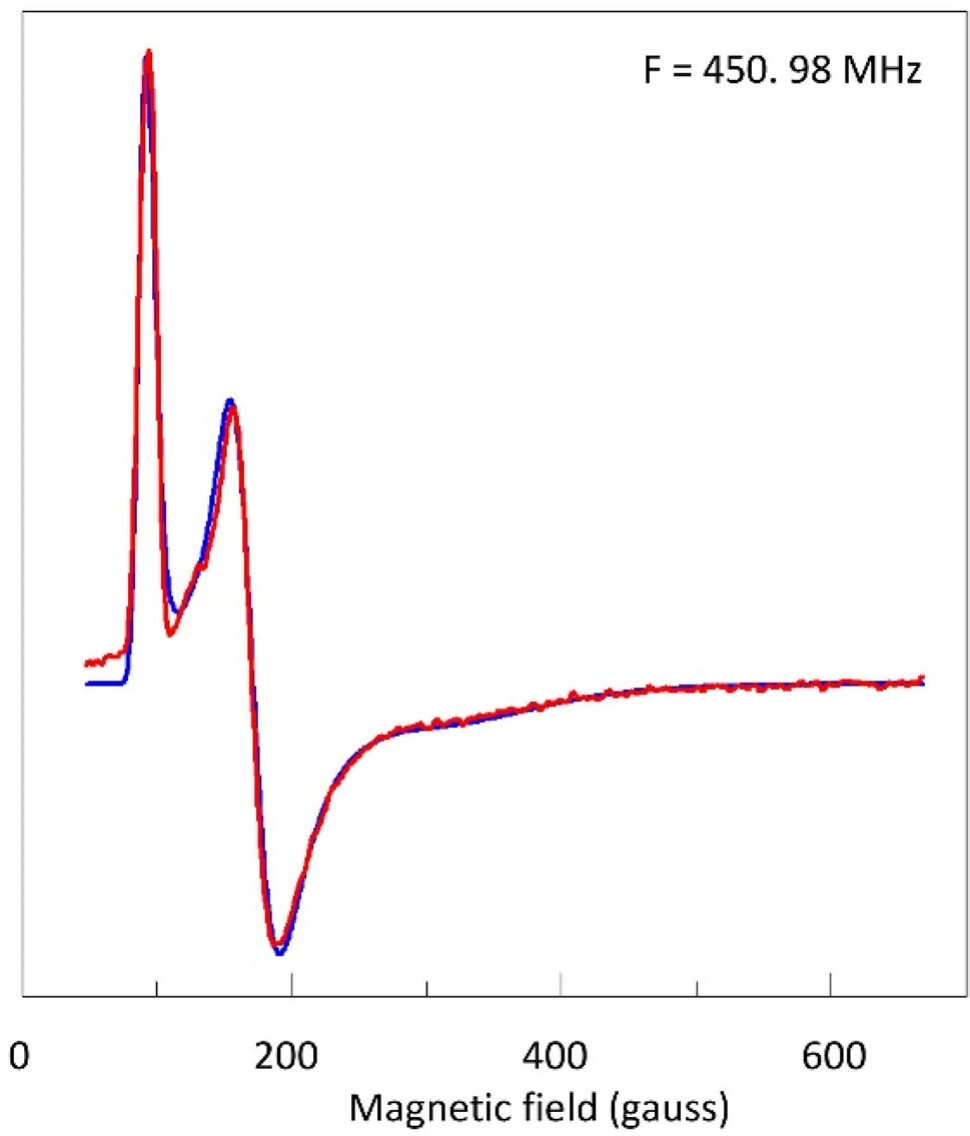
Low-frequency data and simulations of the complete HALS spectrum of myoglobin cyanide. EPR conditions: microwave frequency, 450.98 MHz; incident microwave power, 12 dBm; modulation amplitude, 5 gauss; modulation frequency, 100 kHz; temperature, 9.3 K. Simulation parameters*: g*_*zyx*_ = 3.45, 1.84, 0.70; *W*_*zz,yy,xx*_ = 0.26, 0.17, 0.20 in *g*-value units

At frequencies below some 1 GHz spectra of low-spin hemoproteins begin to broaden due to intermolecular dipole–dipole interactions [11], and since this is a broadening in magnetic-field units, its effect is greatest on the sharpest feature, that is the *g*_*z*_ line. This effect is clearly visible in the spectrum of myoglobin cyanide at 0.451 GHz (Fig. 11), where the *g*_*z*_ line is significantly broadened compared to the rest of the spectrum, leading to an apparent amplification of other features.

Furthermore, compared to X-band the spectrum now extends over a drastically reduced field scale and the whole spectrum is easily covered with a conventional X-band magnet. Visual inspection now readily reveals the *g*_*x*_ line, and the spectrum is unequivocally fit with *g* values 3.45, 1.84, 0.7. From this set the crystal field parameters in Eqs. (9)–(10) can be obtained for interpretation in terms of structure and function. The *S*_*g*_ is 15.78, which is close, but not equal, to 16.00, where the latter would require a *g*_*x*_ = 0.84. This indicates that the generally made assumption for HALS heme spectra of *k* = 1 and *S*_*g*_ = 16 in this case is not rigorously met, and that the subject of low-frequency HALS spectra deserves further investigation.

## Conclusions

I have shown that the novel low-frequency broadband spectroscopy is a useful complementary method in advanced EPR analysis of ferric hemoproteins. In the example of high-spin ferrimyoglobin fluoride broadband EPR completely resolves the ^19^F splitting that is only resolved in one direction in X-band. Inspection of linewidth as a function of frequency allows for a deconvolution of broadening mechanism that would not have been possible on basis of X-band spectra. The EPR of *S* = 5/2 hemoproteins is found to be remarkably insensitive to broadening by intermolecular dipole–dipole interaction.

HALS hemoprotein X-band spectra are notoriously difficult to measure and to analyze because of their broad lines and because they extend beyond the maximal field limit of X-band spectrometers. Since the *g*_*x*_ peak is usually not detectable, but is required for a crystal-field analysis, the assumption is made that the sum of the squares of the *g* values equals 16. As shown with the example of low-spin ferrimyoglobin cyanide, at low frequency the sum-equals-16 rule may not be met, which implies that through these experiments new science may be learned as the complete spectrum is now readily detectable and all *g* values are available for analysis.

## Supplementary Information

Below is the link to the electronic supplementary material.

Supplementary file1 (PDF 661 KB)
