# Supplementary material for: Low-frequency EPR of ferrimyoglobin fluoride and ferrimyoglobin cyanide: a case study on the applicability of broadband analysis to high-spin hemoproteins and to HALS hemoproteins
Source: J Biol Inorg Chem. 2022 Jul 8;27(4-5):497–507. doi: 10.1007/s00775-022-01948-1 (PMC9399021; doi:10.1007/s00775-022-01948-1)

SUPPLEMENTARY INFORMATION for

Low-frequency EPR of ferrimyoglobin fluoride and ferrimyoglobin cyanide: a case study on the applicability of broadband analysis to high-spin hemoproteins and to HALS hemoproteins

Journal of Biological Inorganic Chemistry

Wilfred R. Hagen
Delft University of Technology, Department of Biotechnology, Delft, The Netherlands
w.r.hagen@tudelft.nl

Figure S1. Very low frequency EPR spectrum of myoglobin fluoride showing strength equivalence of superhyperfine interaction and electronic Zeeman interaction. At this low frequency of 0.28 GHz the splitting of the ^{19}F doublet around $g_{||}$ = 2 has become highly asymmetric to the extent that its left-hand negative peak has convoluted with the right-hand feature of the doublet around $g_{\perp}$ = 5.97. Under these conditions proper simulation would require diagonalization of the 12 $\times$ 12 energy matrix for S = 5/2 and I = 1/2, however, simulation assuming the hyperfine interaction to be a perturbation up to second order can be seen to still afford a 'borderline' fit to the experimental spectrum. EPR conditions (black trace): modulation amplitude, 6 gauss; modulation frequency, 100 kHz, incident microwave power, 12 dBm; data collection time, 35 min; temperature, 9.2 K. Simulation parameters (red trace): g_z = 2.00, $g_{\perp}$ = 5.97, A_z = 45.2, $A_{\perp}$ = 22, W_z (FWHH) = 16 gauss, $W_{\perp}$ = 22 gauss.

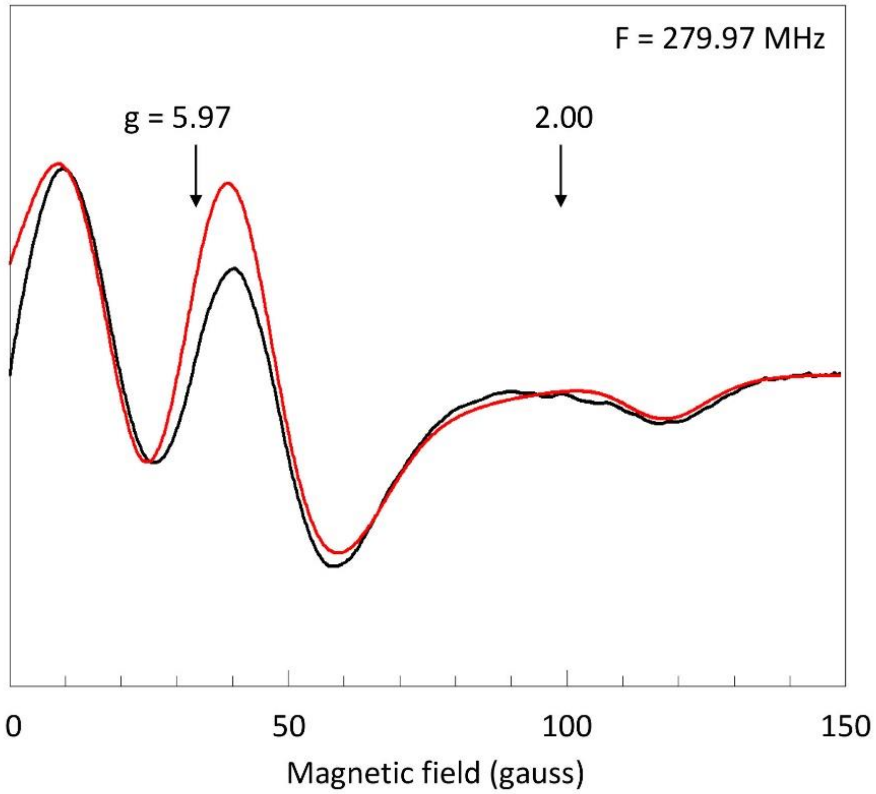

Figure S2. A minor Cu(II) contaminant (< 1%) in myoglobin fluoride. The high-field part of the X-band spectrum of myoglobin fluoride recorded at low microwave power (black and blue traces), is simulated (red and green traces) as a sum of MbF and an axial Cu(II) complex. MbF simulation parameters (*z*, *y*, *x*): *g* = 2.0039, 6, 6; *W* = 9, 9, 9 gauss (HWHH), $A(^{19}F)$ = 45, 24, 24 gauss. Cu(II) simulation parameters: *g* = 2.265, 2.09, 2.09; *W* = 20, 60, 60; $A(^{63,65}Cu)$ = 132, 25, 25. The concentration ratio of MbF and Cu(II() is 100:1. The lowest Kramers doublet of MbF is ca 78 % populated at T = 12 K (when *D* = 5.5 cm^{-1}), therefore, [Cu(II)]/[MbF] < 0.01. EPR conditions: modulation amplitude, 5 gauss; microwave power, 0.2 mW; temperature, 12 K.

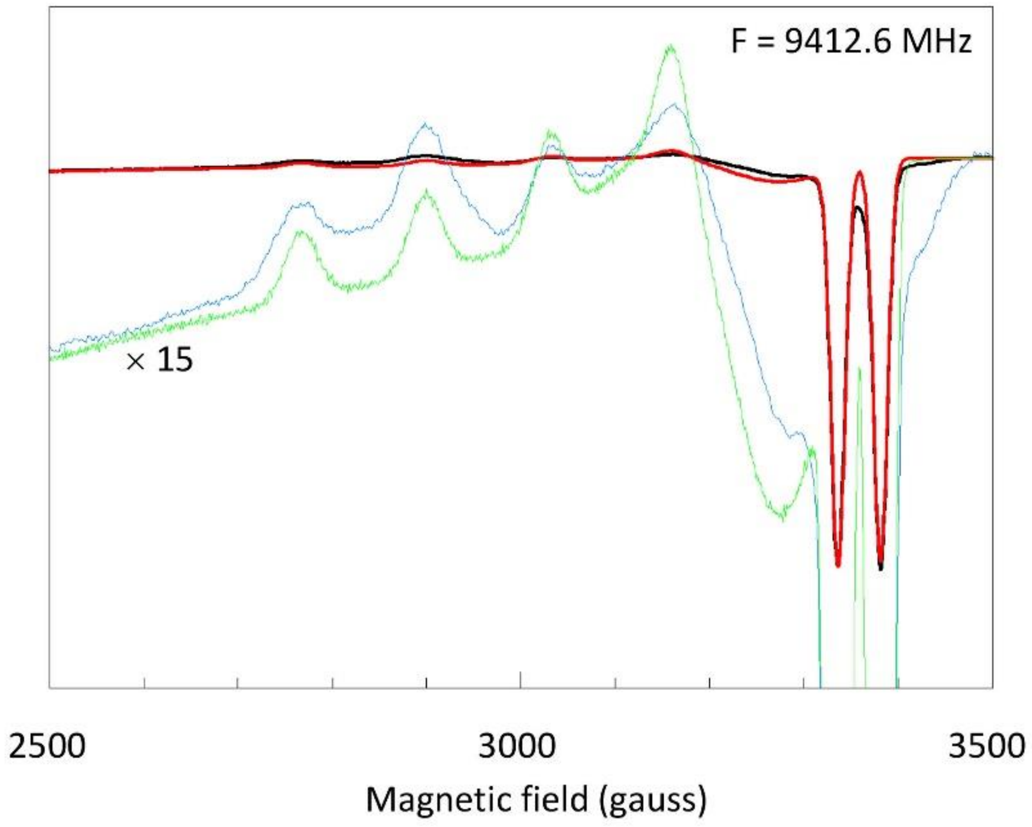

Figure S3. Broadening of the parallel feature in the ERP of myoglobin fluoride is not decreased with reduced protein concentration and reduced modulation amplitude. The red trace is the experimental spectrum of Fig. 8. In the blue trace the concentration of MbF is reduced tenfold from 4 to 0.4 mM and the modulation amplitude is reduced fivefold from 5 to 1 gauss. Invariance of the broadening indicated absence of overmodulation and insignificant broadening due to intermolecular dipolar interaction.

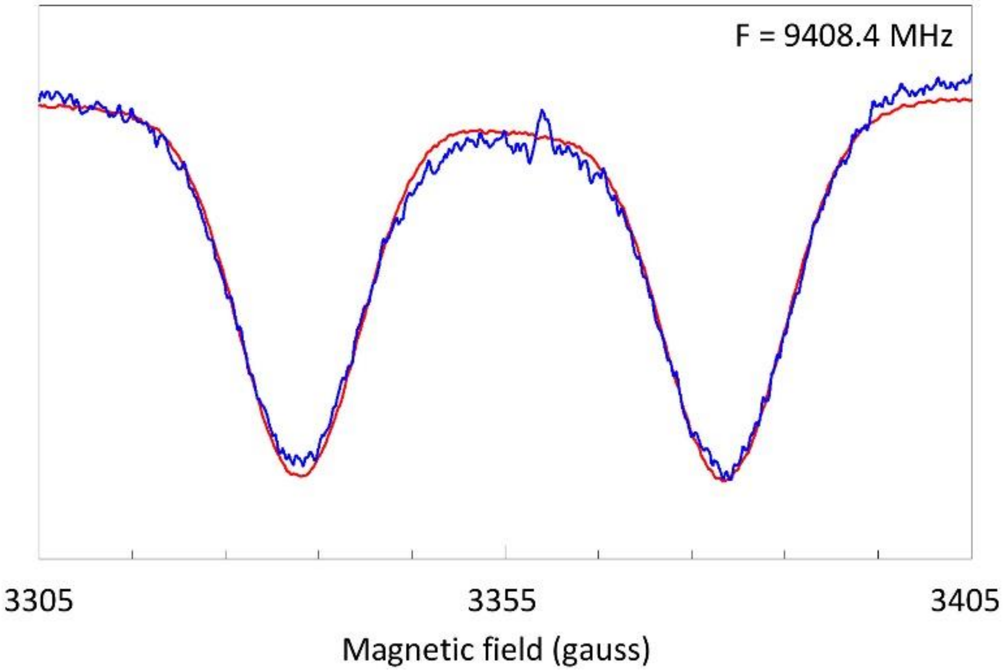

Supplement: Supplementary file 1 — Supplementary file1 (PDF 661 KB) [file 775_2022_1948_MOESM1_ESM.pdf]
